# Evaluating the Immediate Impact of Graphic Messages for Vaping Prevention among Black and Latino Adolescents: A Randomized Controlled Trial

**DOI:** 10.3390/ijerph191610026

**Published:** 2022-08-14

**Authors:** Francisco Cartujano-Barrera, Ruthmarie Hernández-Torrez, Xueya Cai, Rafael H. Orfin, Chiamaka Azogini, Arlette Chávez-Iñiguez, Edgar Santa Cruz, Maansi Bansal-Travers, Karen M. Wilson, Scott McIntosh, Deborah J. Ossip, Ana Paula Cupertino

**Affiliations:** 1 Department of Public Health Sciences, University of Rochester Medical Center, Rochester, NY 14642, USA; 2 Department of Biostatistics and Computational Biology, University of Rochester Medical Center, Rochester, NY 14642, USA; 3 Social Work Program, Cameron Community Ministries, Rochester, NY 14606, USA; 4 Department of Health Behavior, Roswell Park Comprehensive Cancer Center, Buffalo, NY 14263, USA; 5 Department of Pediatrics, University of Rochester Medical Center, Rochester, NY 14642, USA

**Keywords:** electronic cigarettes, e-cigarettes, vaping, adolescents, Black adolescents, Latino adolescents, graphic messages

## Abstract

The purpose of this pilot study was to assess the immediate impact of vaping prevention graphic messages on the susceptibility to future vaping among Black and Latino adolescents (ages 12 to 17). Graphic messages (available in English and Spanish) were developed using participatory research procedures with Black and Latino adolescents. Recruitment was conducted by a team of diverse, bilingual (English and Spanish), trained recruiters. Participants (*n* = 362) were randomized in a 1:1:1:1 schema to receive one of four graphic messages (health rewards, financial rewards, autonomy, and social norms). Overall, all graphic messages but one showed a slight decrease in the number of participants susceptible to future vaping, though none of these differences was statistically significant. The graphic message on health rewards decreased the number of participants susceptible to future vaping the most (55.7% vs. 50%, at pre- vs. post-viewing, *p* = 0.125), followed by the graphic messages on social norms and autonomy (55.1% vs. 52.8%, *p* = 0.687; 55.4% vs. 52.2%, *p* = 0.435; respectively). The graphic message on financial rewards increased the number of participants susceptible to future vaping slightly (52.7% vs. 53.8%, *p* = 1.00). Future research is needed to evaluate susceptibility to future vaping before and after exposure to different and/or repeated vaping prevention graphic messages.

## 1. Introduction

The rapidly increasing popularity of electronic cigarettes (e-cigarettes) has reversed decades of decreasing nicotine and tobacco use among adolescents. According to the U.S. National Youth Tobacco Surveys, in 2021, 34.0% of high school students (an estimated 5.22 million) and 11.3% of middle school students (an estimated 1.34 million) reported ever use of any nicotine and tobacco product [1]. E-cigarettes were the most commonly used nicotine product among high school (11.3%) and middle school (2.8%) students [1]. E-cigarettes have completely transformed the landscape of nicotine use in youth, combining advanced technology, attractive design, and flavors, fueled by aggressive marketing and social media promotion [2,3]. There is robust evidence that e-cigarette use (vaping) during adolescence is associated with future initiation of cigarette, alcohol, and marijuana use [4,5,6]. Moreover, early nicotine exposure puts adolescents at risk for a lifetime of vaping addiction as well as unknown health risks of long-term vaping. Particulate, chemical, and heavy metal exposure from e-cigarettes and risk of acute injuries and toxicity are a public health concern [7,8,9,10,11,12]. Vaping has been connected to 2807 lung injury cases and 68 deaths in the US (as of February 2020) [13].

Despite the adverse health effects and high prevalence of vaping among adolescents, there are a lack of effective messages and communication channels to prevent initiation. To date, we know little about whether messages can prevent vaping among adolescents and, if so, what messages and delivery formats may be most effective. Very little is also known about the differential effects of these messages on different vulnerable and underserved communities (e.g., adolescents, racial/ethnic minority groups). One qualitative study with 159 adolescents (21% Black and 9% Hispanic) found that messages focusing on addiction alone did not resonate with participants [14]. Participants wanted more information about negative consequences of vaping [14]. One quantitative study with 563 adolescents (7.6% Black and 14.2% Hispanic) found that, compared to gain-framed text messages, loss-framed messages were more effective to dissuade at-risk youth from vaping [15]. One limitation of both studies is the underrepresentation of Black and/or Latino adolescents. This leaves a substantial gap in communication research for vaping prevention among racial and ethnic minority groups. A qualitative study with 63 adolescents (52% Black and 6% Hispanic) and 27 parents (89% Black, no reported data on ethnicity) noted that participants perceived a lack of racial and ethnic diversity in existing e-cigarette prevention campaigns [16]. Moreover, youth participants disliked ads that looked like “an older person made it for teenagers” [16]. The present study assesses the immediate impact of vaping prevention graphic messages on the susceptibility of future vaping among Black and Latino adolescents. Graphic messages were developed using participatory research procedures with Black and Latino adolescents [17].

## 2. Materials and Methods

### 2.1. Study Design

We conducted a pilot randomized controlled trial (RCT) to assess the immediate impact of vaping prevention graphic messages on the susceptibility of future vaping among 362 Black and Latino adolescents, with equal representation between the two groups. The primary outcome was the change in susceptibility to future vaping before and after exposure to the graphic messages. The study design and implementation were informed by a Community Advisory Board of Black and Latino adolescents. Study procedures were approved and monitored by the University of Rochester Medical Center Institutional Review Board (STUDY00006267) and registered on ClinicalTrials.gov (NCT04899999). Participants were compensated with a $25 gift card for their time and effort.

### 2.2. Conceptual Framework

The biobehavioral model of nicotine addiction was used as the framework for graphic message development and interpretation [17]. The biobehavioral model of nicotine addiction recognizes the influence of social (e.g., social norms), psychological (e.g., rewards, autonomy), and biological factors in relation to nicotine and tobacco use [18]. Moreover, this study is supported by and builds upon our established history of using social cognitive theory for tobacco control research [19,20,21,22].

### 2.3. Recruitment

Recruitment was conducted by a team of diverse, bilingual (English and Spanish), trained recruiters between August and December 2021. Proactive recruitment strategies included study presentations at community-based events (e.g., festivals, health fairs), school-based events (e.g., back to school events, after-school programs), and recreational centers (e.g., fitness centers, malls) [23]. Reactive recruitment strategies included study advertisements via social media (e.g., Facebook posts shared by local community-based organizations), word of mouth, and an academic-based research hub (i.e., UR Health Research—an institutional resource to promote participation in clinical trials) [23]. The details of the recruitment strategies are described thoroughly in a previous publication [23].

### 2.4. Eligibility

Individuals were eligible if they (1) self-identified as African American/Black and/or Hispanic/Latino, (2) knew how to read and speak English and/or Spanish, (3) were at least 12, but not greater than 17 years old, (4) had never used e-cigarettes, and (5) had access to a device that would allow them to connect to the online survey (e.g., desktop, laptop, tablet, and/or smartphone).

### 2.5. Screening and Consent

Trained research staff determined participant eligibility. Eligible adolescents and their parents/guardians received informational letters describing the study, explaining the risks as well as benefits, and outlining the team contact information. Once the parents/guardians and adolescents reviewed the informational letter, trained research staff obtained parent/guardian permission and adolescent assent. Eligibility assessment and consent were available in each parent’s/guardian’s and adolescent’s language of preference, either English or Spanish. The screening and consent procedures are described in detail in a previous publication [23].

### 2.6. Randomization

After providing informed consent and completing baseline measures, participants were randomized in a 1:1:1:1 schema to receive one of four graphic messages (health rewards, financial rewards, autonomy, and social norms). Randomization occurred at the participant level using the on-board randomization module in the Research Electronic Data Capture (REDCap) database system [24]. Neither the participant nor study staff knew in advance the group assignment of each participant.

### 2.7. Intervention

All graphic messages were developed in English and Spanish using participatory research procedures with Black and Latino adolescents. Importantly, all graphic messages portrayed Black and Latino adolescents. The development and description of the graphic messages have been detailed elsewhere [17]. The graphic messages incorporated four main theoretical constructs: health rewards, financial rewards, autonomy, and social norms [17].

#### 2.7.1. Health Rewards

The graphic message showed a mother kissing her son who is hospitalized in the intensive care unit due to a lung injury related to vaping. The caption was “Dying for a vape? It hurts more than you know”.

#### 2.7.2. Financial Rewards

The graphic message showed a teenage boy looking at a piggy bank, wondering where his money went. In addition, the piggy bank had e-cigarette aerosol coming out of its mouth and an e-cigarette falling out of it. The caption read “Vaping leads to nothing. Don’t let your money vaporize away”.

#### 2.7.3. Autonomy

The graphic message showed Black and Latino adolescents being targeted by a sniper’s scope. The caption was “Vaping companies are targeting Black and Latino teens. Your life matters. Don’t let them take it away”.

#### 2.7.4. Social Norms

The graphic message showed a group of Black and Latino teens standing together, with some holding e-cigarettes in their hands and e-cigarette aerosol covering their faces. The graphic message also included two teens without e-cigarettes, but with smoke around their faces. The caption was “Just because vaping is common doesn’t mean it’s cool. Stay woke. Don’t smoke”.

### 2.8. Assessments

All assessments were completed in the participants’ language of preference, either English or Spanish. Assessments were adapted from surveys used in previous studies and pre-tested for survey administration among the research team [25]. The baseline survey collected information on demographics (e.g., race, ethnicity, age, gender, sexual orientation, state of residence, and employment status). Participants’ state of residence was grouped into one of five regions (e.g., Northeast, Midwest, South, West, and Puerto Rico), in accordance with the U.S. Census Bureau geographical map [26].

The primary outcome was the change in susceptibility to future vaping before and after exposure to the graphic messages. Informed by prior foundational research on youth electronic cigarette use, susceptibility to future vaping was assessed with three items tapping curiosity, intent, and social influence [25]. Participants were asked: “Have you ever been curious about using e-cigarettes/vaping?”; “Do you think that you will use e-cigarettes/vape in the next 12 months?”; and “If one of your best friends were to offer you an e-cigarette/electronic vapor product, would you use it?” (1 = “Definitely not” to 4 = “Definitely yes”). These response categories were combined to create dichotomous variables (1 = “Definitely not”; 2 = “Probably not”, “Probably yes”, and “Definitely yes”). Participants who responded with a response other than “Definitely not” to one or more items were deemed susceptible (Yes/No). Susceptibility to future vaping was selected as the primary outcome of the study because of its robust predictive validity [27,28,29].

The secondary outcome was participant satisfaction with the graphic messages. Satisfaction measures included questions such as “How satisfied are you with the image?” (1 = “Extremely unsatisfied” to 5 = “Extremely satisfied”) and “Would you recommend this picture to a friend?” (Yes/No).

### 2.9. Sample

This was a pilot study to detect differences in susceptibility to future vaping before and after exposure to the graphic messages. A convenient sample of 362 participants was used in the study to estimate the effect size for future studies.

### 2.10. Analyses

Characteristics of enrolled participants were summarized with percentages for categorical variables, and with means and standard deviations (SD) for continuous variables. Cronbach’s alpha was used to assess the internal consistency reliability of the susceptibility to future vaping questionnaire. To evaluate randomization success, participants’ age differences between groups were compared with a one-way ANOVA test. Moreover, categorical variables (e.g., gender, sexual preference, race/ethnicity, region, language of preference, employment, grade, recruitment type, and baseline susceptibility to future vaping) were compared between groups using Chi-square tests. The McNemar test was used to determine if there were differences between pre- and post-assessment on susceptibility to future vaping. All analyses were performed in SPSS 14.0, a statistical software developed by IBM ( Armonk, NY, USA).

## 3. Results

**Figure 1:**
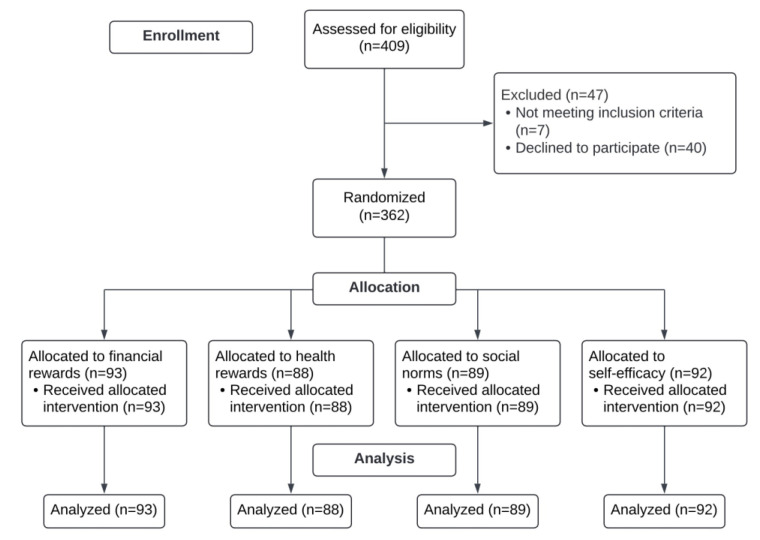
CONSORT flow diagram.

A total of 409 individuals were assessed for study eligibility; 402 (98.2%) met the eligibility criteria. Overall, 362 adolescents consented to participate in the RCT and were enrolled in the study (Figure 1). The results of the recruitment strategies are described thoroughly in a previous publication [23].

Randomization resulted in similar baseline characteristics between intervention groups (Table 1). Participants were on average 15 years old (SD 1.49), 64.9% were male, 87.6% were heterosexual or straight, 50% were Hispanic/Latino, and 50% were Black/African American. Two thirds (66.3%) of participants lived in the Northeast region of the U.S., 82.0% reported English being their language of preference, and 18.8% were currently employed. One quarter (25.1%) of participants were in 11th grade, 56.4% were recruited via reactive methods, and 54.7% were susceptible to future vaping. Notably, the Cronbach’s alpha coefficient for the susceptibility to future vaping questionnaire was 0.848.

Overall, all graphic messages but one showed a slight decrease in the number of participants susceptible to future vaping, though none of these differences was statistically significant (Table 2). The graphic message on health rewards decreased the number of participants susceptible to future vaping the most (55.7% vs. 50%, at pre- vs. post-viewing, *p* = 0.125), followed by the graphic messages on social norms and autonomy (55.1% vs. 52.8%, *p* = 0.687; 55.4% vs. 52.2%, *p* = 0.435; respectively). The graphic message on financial rewards increased the number of participants susceptible to future vaping slightly (52.7% vs. 53.8%, *p* = 1.00).

The graphic message on health rewards resulted in the highest number of participants who reported being satisfied or very satisfied with the graphic message (69.3%, 61/88) and would recommend the graphic message to a friend (94.3%, 83/88). The graphic message on financial rewards had a similar result, with 68.8% of participants reporting being satisfied or very satisfied with the graphic message and 90.3% reporting that they would recommend the graphic message to a friend. The graphic message on autonomy had the lowest satisfaction, with only 34.7% of participants reporting being satisfied or very satisfied and 70.6% that they would recommend the graphic message to a friend.

## 4. Discussion

To the best of our knowledge, this is the first study to assess the immediate impact of vaping prevention graphic messages on susceptibility to future vaping among Black and Latino adolescents. This study is timely given that Black and Latino adolescents are often underrepresented in tobacco control studies, and the results of this study suggest that 55% of them are susceptible to future vaping. Notably, the graphic messages were developed by Black and Latino adolescents using a qualitative, user-centered design method [17]. Moreover, the study design and implementation were informed by a Community Advisory Board of Black and Latino adolescents. Involvement of Black and Latino adolescents was critical in the development of the intervention messages to ensure appropriate and impactful words and images were used to communicate the risks of vaping.

This is the first study from our literature review to evaluate the change in number of participants susceptible to future vaping before and after exposure to graphic messages as the primary outcome. There is robust evidence that susceptibility to future vaping prospectively predicts e-cigarette use behavior [27,28,29]. Moreover, this study revealed that the susceptibility to future vaping questionnaire has an acceptable internal consistency. In this study, the changes in the number of participants susceptible to future vaping were quite small and not statistically significant. However, this small effect may be expected from a one-time exposure to the graphic messages. Additional research is needed to evaluate susceptibility to future vaping before and after exposure to different and/or repeated vaping prevention graphic messages. Moreover, future research should evaluate the population impact of a small reduction in the number of adolescents susceptible to future vaping. Findings from this study may also be helpful in the development of effective graphic images for health warning labels on electronic cigarette packages.

In this study, the graphic message on health rewards decreased the number of participants susceptible to future vaping the most and resulted in the highest number of participants who reported being satisfied or very satisfied with the graphic message and that would recommend the graphic message to a friend. This result is consistent with a previous online RCT with 928 adolescents (81% white, 90% non-Hispanic) that aimed to evaluate social media messages designed to educate on the topic of e-cigarettes [30]. In that RCT, “messages that communicate non-addiction health effects, especially for harms with social implications, had the greatest intended effects (e.g., increased unpleasantness of vaping) among adolescents... Intended message reactions were strongest for topics about missing out because of lung damage…” [30].

The graphic messages on social norms and autonomy similarly decreased the number of participants susceptible to future vaping. In contrast, the graphic message on financial rewards had the opposite effect as it increased the number of participants susceptible to future vaping. This result is surprising as financial rewards have been used in an attempt to reinforce and sustain behavior, including smoking initiation among youth [31]. However, this result echoes those from a qualitative study where one ad focused on the financial costs of vaping generated mixed reactions (e.g., some participants felt the ad was not accurate because e-cigarettes are cheap, especially when users refill their own pods) [32]. The shareability of electronic cigarettes as well as the consistent increase in online distribution (e.g., sales, promotions, bulk purchases) could have led to a dulling of impact on the basis of financial rewards [25,32]. Moreover, the degree of financial dependence of adolescents on their parent/caregiver should be taken into consideration in the future when considering the utilization of financial rewards as a means of prevention.

Importantly, four of five adolescents (83.9%) indicated that they would share the graphic message with a friend. Designing messages in a manner that is consistent with the adolescents’ interest may increase their desire to share with friends, which could be instrumental to organically increasing peer-to-peer message reach. In the context of e-cigarette and cigarette warnings, sharing messages not only increased the reach of the messages but also spark social interactions about the health harms of tobacco or the benefits of quitting that may act as a social intervention reaching beyond the individual [33,34].

### Strengths and Weaknesses

This study adds valuable insights to the expanding literature on vaping prevention. Study results demonstrate that it is feasible to recruit Black and Latino adolescents—two traditionally hard-to-reach groups—into a vaping prevention RCT. Furthermore, tobacco prevention research and programs typically only focus on at-risk youth (e.g., individuals susceptible to future tobacco use) [15,35]. In contrast, this study did not limit the investigation to at-risk youth in order to maximize study participation and relevance of findings to future intervention efforts. Another study strength is the use of graphic messages that were developed by Black and Latino adolescents using a qualitative, user-centered design method [17]. The inclusion of Spanish-speaking participants is another study strength—18% of participants in this study selected Spanish as their language of preference.

The limitations of this study need to be acknowledged. First, this was a pilot study with a modest sample size, which decreases the power to detect differences pre- and post-exposure to the graphic messages. Second, as a one-time study without follow-up assessments, we were unable to track actual youth vaping behavior. Although this design is typical of message pretesting studies, it nonetheless cautions against extrapolating the current findings to a potential campaign effect on behavior change [35]. It is imperative to examine behavior in a longitudinal design as an outcome of vaping prevention graphic messages. Third, this study only examined susceptibility to future vaping among never users as an outcome. A sharp focus on this outcome is justifiable given prior foundational research on youth electronic cigarette use. Nevertheless, a wider range of outcomes (e.g., e-cigarette cravings and e-cigarette risk beliefs) would probably improve the current ability to fully capture the potential impact of the graphic messages under investigation. Fourth, the study did not capture how participants accessed the survey or the graphic messages (e.g., via their desktop, laptop, tablet, and/or smartphone). As a consequence, it is unknown if the immediate impact of these graphic messages varies by type of access device. Lastly, this study only evaluated one graphic message per theoretical construct, limiting the ability to assess the immediate impact of each theoretical construct.

## 5. Conclusions

The changes in number of Black and Latino participants susceptible to future vaping before and after exposure to the graphic messages were quite small and not statistically significant. The graphic message on health rewards decreased the number of participants susceptible to future vaping the most and resulted in the highest number of participants who reported being satisfied or very satisfied with the graphic message and that would recommend the graphic message to a friend. The graphic messages on social norms and autonomy similarly decreased the number of participants susceptible to future vaping. In contrast, the graphic message on financial rewards had the opposite effect, as it increased the number of participants susceptible to future vaping. Future research is needed to evaluate susceptibility to future vaping before and after exposure to different and/or repeated vaping prevention graphic messages.

## Figures and Tables

**Table 1:** Sociodemographic characteristics of participants.

Characteristic	Full Sample *n* = 362		Financial Rewards *n* = 93		Health Rewards *n* = 88		Social Norms *n* = 89		Autonomy *n* = 92		*p*-Value
	**M**	**SD**	**M**	**SD**	**M**	**SD**	**M**	**SD**	**M**	**SD**	
*Age*	15.00	1.49	15.04	1.61	15.06	1.35	14.89	1.52	15.00	1.46	0.872
	***n***	**%**	***n***	**%**	***n***	**%**	***n***	**%**	***n***	**%**	
*Gender*											
Male	235	64.9	54	58.1	54	61.4	62	69.7	65	70.7	0.313
Female	124	34.3	38	40.9	34	38.6	26	29.2	26	28.3	
Transgender Male	1	0.3	1	1.1	-	-	-	-	-	-	
Transgender Female	1	0.3	-	-	-	-	1	1.1	-	-	
Gender variant/non-conforming	1	0.3	-	-	-	-	-	-	1	1.1	
*Sexual Preference*											
Heterosexual/straight	317	87.6	81	87.1	81	92.0	82	92.1	73	79.3	0.070
Homosexual/gay	6	1.7	-	-	1	1.1	1	1.1	4	4.3	
Bisexual	13	3.6	4	4.3	1	1.1	5	5.6	3	3.3	
Not listed	7	1.9	3	3.2	1	1.1	-	-	3	3.3	
Prefer not answer	19	5.2	5	5.4	4	4.5	1	1.1	9	9.8	
*Race/ethnicity*											
Hispanic/Latino	181	50	45	48.4	46	52.3	44	49.4	46	50.0	0.962
Black/African American	181	50	48	51.6	42	47.7	45	50.6	46	50.0	
*Region*											
Northeast	240	66.3	65	69.9	53	60.2	58	65.2	64	69.6	0.238
West	52	14.4	13	14.0	15	17.0	15	16.9	9	9.8	
Midwest	11	3.0	-	-	2	2.3	6	6.7	3	3.3	
South	41	11.3	11	11.8	11	12.5	9	10.1	10	10.9	
Puerto Rico	18	5.0	4	4.3	7	8.0	1	1.1	6	6.5	
*Language of preference*											
Spanish	65	18.0	15	16.1	17	19.3	14	15.7	19	20.7	0.783
English	297	82.0	78	83.9	71	80.7	75	84.3	73	79.3	
*Currently employed*											
Yes	68	18.8	28	19.4	19	21.6	16	18.0	15	16.3	0.830
No	294	81.2	75	80.6	69	78.4	73	82.0	77	83.7	
*Grade*											
6th	4	1.1	2	2.2	-	-	-	-	2	2.2	0.393
7th	24	6.6	9	9.7	5	5.7	6	6.7	4	4.3	
8th	43	11.9	9	9.7	7	8.0	11	12.4	16	17.4	
9th	61	16.9	14	15.1	19	21.6	18	20.2	10	10.9	
10th	82	22.7	21	22.6	17	19.3	21	23.6	23	25.0	
11th	91	25.1	22	23.7	29	33.0	17	19.1	23	25.0	
12th	57	15.7	16	17.2	11	12.5	16	18.0	14	15.2	
*Recruitment*											
Proactive	158	43.6	39	41.9	35	39.8	40	44.9	44	47.8	0.714
Reactive	204	56.4	54	58.1	53	60.2	49	55.1	48	52.2	
*Susceptible to future vaping*											
Yes	198	54.7	49	52.7	49	55.7	49	55.1	51	55.4	0.976
No	164	45.3	44	47.3	39	44.3	40	44.9	41	44.6	

**Table 2:** Pre- and post-exposure on susceptibility to future vaping.

Characteristic	Full Sample *n* = 362	Financial Rewards *n* = 93	Health Rewards *n* = 88	Social Norms *n* = 89	Autonomy *n* = 92
Pre-exposure, *n* (%)	198 (54.7%)	49 (52.7%)	49 (55.7%)	49 (55.1%)	51 (55.4%)
Post-exposure, *n* (%)	189 (52.2%)	50 (53.8%)	44 (50%)	47 (52.8%)	48 (52.2%)
*p*-value	0.733	1.000	0.125	0.687	0.435
% of change	−4.6%	+2.1%	−10.2%	−4.2%	−5.8%
Satisfied/Very satisfied, *n* (%)	207 (57.1%)	64 (68.8%)	61 (69.3%)	50 (56.1%)	32 (34.7%)
Recommend to a friend, *n* (%)	304 (83.9%)	84 (90.3%)	83 (94.3%)	72 (80.8%)	65 (70.6%)

## Data Availability

The datasets generated for this study are available on request to the corresponding author.
